# Artificial Intelligence for Clinical Decision Support in Internal Medicine: A Systematic Review

**DOI:** 10.7759/cureus.115568

**Published:** 2026-08-31

**Authors:** Muhammad Usman Taj, Ahmed Abdelsalam Mohamed Osman, Fatmaalzahra Ahmed Lotfey Habib, Safwa Obeid Eltreifi Obeid, Nada Omer Mokhtar Babkir, Lubna Mohammed Jabier Abdullah, Hiba Elawad Ali Mohamed

**Affiliations:** 1 Intensive Care Unit, University Hospital Sharjah, Sharjah, ARE; 2 Gastroenterology and Hepatology, Southport and Ormskirk Hospital NHS Trust, Southport, GBR; 3 Nephrology, Mater Misericordiae University Hospital, Dublin, IRL; 4 Emergency Medicine, Dr Suleiman Al Habib Hospital, Olaya, SAU; 5 General Practice, Medical City for Military and Security Services, Muscat, OMN; 6 Emergency Medicine, Hotat Bani Tamim General Hospital, Riyadh, SAU

**Keywords:** artificial intelligence, clinical decision support systems, electrocardiography, internal medicine, machine learning, sepsis, systematic review

## Abstract

Artificial intelligence (AI) models perform well on retrospective internal medicine datasets, but few have been deployed prospectively as clinician-facing decision support and evaluated for their effect on care. The purpose of this study was to synthesise original primary research evaluating prospectively deployed AI-based clinical decision support systems (CDSSs) in adult internal medicine and acute general medical care.

Five databases were searched from inception to 31 May 2026 for original peer-reviewed studies in which an AI-derived CDSS was deployed in a live clinical workflow and compared with usual care or a pre-implementation period. Non-original publications and retrospective model development or validation studies were excluded. Screening, extraction and risk-of-bias assessment (RoB 2; ROBINS-I) were performed in duplicate. Heterogeneity precluded meta-analysis, so findings were synthesised structurally.

Nine studies (2020-2025) were included: six randomised trials and three non-randomised implementation studies from the United States (n = 6), Taiwan (n = 2) and Thailand (n = 1), contributing 110,452 patients plus one clinician-level trial of 50 physicians. Four domains emerged: sepsis (four studies), inpatient deterioration (one), AI-enabled electrocardiography (three) and large language model-assisted diagnostic reasoning (one). Process outcomes improved consistently, including shorter time to antibiotics, higher sepsis bundle compliance, more new diagnoses of low ejection fraction and better diagnostic yield of confirmatory testing. A mortality benefit was reported in four studies, but three of these were non-randomised, and in the only randomised trial with mortality as its primary endpoint the effect was concentrated in an algorithm-defined high-risk subgroup. The single large language model trial showed no improvement in diagnostic reasoning. Three randomised trials were at low risk of bias, two raised some concerns and one was at high risk; two non-randomised studies were at moderate and one at serious risk of bias.

Deployed AI-based CDSSs reliably improve internal medicine care processes, but evidence for patient-important benefit remains limited, concentrated in sepsis and cardiac phenotyping, and geographically narrow. Benefit appears to depend on how alerts are routed and acted upon as much as on model discrimination. Multi-centre randomised trials with patient-important primary endpoints are required before broad adoption.

## Introduction and background

Internal medicine is defined by diagnostic breadth and by the density of decisions made under uncertainty. Physicians caring for undifferentiated adults must integrate vital signs, laboratory trends, imaging, medication histories and comorbidity burden, often within minutes and frequently at the margins of their own specialty expertise. Computerised clinical decision support systems (CDSSs) were introduced precisely to reduce this cognitive load, and their conceptual foundations, delivery mechanisms and failure modes have been described in detail [1]. However, the aggregate benefit of conventional, rule-based CDSSs has proved modest. A meta-analysis of controlled clinical trials found that computerised decision support produced small to moderate absolute improvements in the processes of care it targeted, with wide variability across settings and only limited translation into patient-level benefit [2]. Rule-based systems are constrained by the very feature that makes them transparent: they encode a fixed, human-specified logic that cannot adapt to the high-dimensional, temporally evolving data streams generated by hospitalised medical patients.

Artificial intelligence (AI), and in particular machine learning (ML) and deep learning, offers a qualitatively different approach. Rather than applying predetermined rules, these models learn representations directly from electronic health record (EHR) data, physiological waveforms and unstructured text, and can therefore detect patterns that are not explicitly programmed and often not clinically obvious [3]. In internal medicine, this capability has been directed at prognosis, diagnosis, treatment selection and workflow efficiency, and the technical basis for these applications is now well established [4]. Three model classes dominate the clinical literature. The first is supervised ML applied to tabular EHR streams, in which vital signs, laboratory values, orders and comorbidity indices are used to generate continuously updated risk estimates for events such as sepsis or clinical deterioration. The second is deep learning, typically a convolutional neural network, applied to raw physiological waveforms, of which the 12-lead electrocardiogram is by far the most extensively developed example. The third is the general-purpose large language model (LLM) applied to unstructured clinical text, which has entered clinical use most recently and is evaluated quite differently from the other two. Yet the maturity of the underlying models has consistently outpaced the maturity of their clinical evaluation. The overwhelming majority of published AI studies in medicine report retrospective discrimination metrics such as the area under the receiver operating characteristic curve (AUROC, a measure of how well a model separates patients who go on to experience an outcome from those who do not, where 0.5 represents chance and 1.0 perfect separation), computed on historical data in which no clinician ever saw the model output and no patient's care was altered. Such studies establish that a signal exists; they cannot establish that surfacing that signal to a physician changes what the physician does, still less that it changes how the patient fares. This distinction defines the scope of the present review. By prospective deployment we mean that the model output was returned to a clinician within a live clinical workflow while the patient was still under care and could still be acted upon; by retrospective validation we mean that a model was applied to archived data that no clinician saw and that no patient's care could have been altered by. Only the former was eligible here.

This translational gap has been quantified repeatedly. Systematic reviews of randomised controlled trials of AI prediction tools have found that only a small minority of published algorithms have ever been evaluated prospectively, that trials are concentrated in a narrow set of specialties, and that reporting quality is frequently inadequate [5,6]. In response, AI-specific reporting standards such as the CONSORT-AI extension have been developed to improve the transparency of trials evaluating AI interventions, including specification of the algorithm version, the input data handling, the human-AI interaction and the handling of poor-quality or out-of-distribution inputs [7]. Despite these developments, no synthesis has focused specifically on internal medicine, the discipline in which the largest share of hospitalised adults receive care and in which the potential impact of decision support is correspondingly greatest. Existing reviews have been organised either around a single condition, such as sepsis, or around AI in medicine as an undifferentiated whole, leaving clinicians and hospital leaders without a consolidated account of what has actually been demonstrated when an AI-based CDSS is switched on in a general medical setting. For the purposes of this review, internal medicine is taken to comprise general medical and step-down wards, hospital medicine and medical subspecialty services, general internal medicine and primary care clinics, and acute care areas in which undifferentiated adult medical presentations are managed; closed intensive care, surgical, obstetric and paediatric settings are outside its scope. The present systematic review was therefore undertaken to identify all original primary research in which an AI-based CDSS was prospectively deployed, in the sense defined above, in adult internal medicine or acute general medical care, to characterise the interventions and their comparators, to summarise their effects on process and patient outcomes, and to appraise the risk of bias in the underlying evidence.

## Review

Materials and methods

Protocol and Reporting

This systematic review was conducted and is reported in accordance with the Preferred Reporting Items for Systematic Reviews and Meta-Analyses (PRISMA) 2020 statement [8]. Eligibility criteria, search strategy, outcomes of interest and the analytical approach were specified a priori in an internal protocol agreed by the review team before screening commenced. The review was not prospectively registered, which is acknowledged as a limitation; the a priori protocol has, however, been deposited in a public repository and is available to readers on request to the corresponding author. As only published, aggregate data were used, ethical approval and informed consent were not required.

Eligibility Criteria 

Eligibility criteria were structured using the Population, Intervention/Exposure, Comparator, Outcomes and Study design (PICOS) framework [9] and are presented in full in Table 1. In brief, eligible studies enrolled adults aged 18 years or older receiving care in an internal medicine context, or clinicians trained in a general medical specialty making decisions for such patients; evaluated a CDSS whose output was generated by an AI or ML method and was prospectively delivered to a clinician within a live clinical workflow; compared this with usual care, a conventional non-AI alerting process, or a contemporaneous or counterfactually modelled pre-implementation period; and reported at least one patient-important, process-of-care or diagnostic outcome. Eligible designs were randomised trials (individual, cluster or stepped-wedge), prospective comparative cohort studies, and quasi-experimental before-and-after studies with a contemporaneous or modelled comparison. Non-original publication types and purely retrospective model development or validation studies in which no clinician received the model output were excluded.

**Table 1:** Eligibility criteria based on the PICOS framework PICOS: Population, Intervention/Exposure, Comparator, Outcomes, Study design; AI: artificial intelligence; ICU: intensive care unit; MEWS: Modified Early Warning Score; NEWS: National Early Warning Score; qSOFA: quick Sequential Organ Failure Assessment; SIRS: systemic inflammatory response syndrome.

PICOS domain	Inclusion criteria	Exclusion criteria
Population (P)	Adults ≥18 years in an internal medicine context: general medical and step-down wards, medical subspecialty services, general internal medicine or primary care clinics, or acute care areas managing undifferentiated adult medical presentations. Clinicians trained in a general medical specialty were eligible participants where the outcome was decision quality.	Paediatric or obstetric populations; exclusively surgical or perioperative populations; closed intensive care populations; non-medical specialty indications; simulated patients without clinician participants.
Intervention/Exposure (I)	A clinical decision support system whose output was generated by artificial intelligence or machine learning (including deep neural networks and large language models) and was prospectively delivered to a clinician, nurse, pharmacist or care team within a live clinical workflow.	Fixed rule-based systems or conventional severity scores (SIRS, qSOFA, MEWS, NEWS) without a learned component; algorithms run silently; models evaluated only on historical data; autonomous devices without a clinician-facing function.
Comparator (C)	Usual care; clinical judgement without access to the algorithm; a conventional non-AI alerting process; a concurrent non-implemented site; or a contemporaneous or counterfactually modelled pre-implementation period.	No comparator group or period; single-arm feasibility, usability or acceptability studies; algorithm-versus-algorithm comparisons without a clinical care comparator.
Outcomes (O)	At least one of patient-important outcomes (mortality, organ dysfunction, ICU transfer, length of stay, days alive and out of hospital); process outcomes (time to antibiotics, bundle compliance, test utilisation, specialist consultation); or diagnostic outcomes (new diagnoses, positive predictive value, diagnostic reasoning quality).	Model discrimination or calibration metrics only; clinician attitudes, trust or satisfaction only; technical implementation metrics only.
Study design (S)	Original peer-reviewed primary research: randomised trials (individual, cluster or stepped-wedge); prospective cohort studies with a defined comparator; quasi-experimental before-and-after studies with a contemporaneous or modelled comparison.	Reviews, systematic reviews and meta-analyses; conference abstracts; editorials, commentaries and letters; case reports and series; protocols; preprints; other non-original publication types.
Other criteria	Full text available; sufficient detail for data extraction and risk-of-bias assessment. No language or geographic restriction. Search period: inception to 31 May 2026.	Full text unobtainable; duplicate publication of the same cohort and outcome (most complete report retained).

Information Sources and Search Strategy

MEDLINE via PubMed, Embase, Scopus, Web of Science Core Collection and the Cochrane Library were searched from inception to 31 May 2026, without language or geographic restriction; because fewer than 11 studies met the eligibility criteria, no additional date restriction was applied. The search combined three Boolean concept blocks covering AI and ML, clinical decision support and alerting, and internal medicine settings and conditions, with controlled vocabulary (MeSH and Emtree) mapped to each database and adapted to its syntax; the complete database-specific search strings are provided in the Appendix. Reference lists of all included articles and of relevant reviews identified during screening were hand-searched, and forward citation tracking was performed in Scopus and Google Scholar. Records were exported to EndNote 21 for deduplication and screened in Rayyan, with two reviewers independently screening titles and abstracts and then assessing full texts; inter-reviewer agreement at the full-text stage was substantial (Cohen's kappa = 0.84), and disagreements were resolved by discussion or by a third reviewer.

Data Extraction

Data were extracted independently and in duplicate onto a piloted, standardised extraction form. Extracted fields comprised bibliographic details (first author, year, country and setting); study design and unit of allocation; participant characteristics and sample size; a technical description of the AI intervention, including model class, input data streams, output format, alert recipient and delivery mechanism, and any reported discrimination or operating characteristics; the nature of the comparator; the prespecified primary and secondary outcomes; and all reported effect estimates with their measures of precision. Where a study reported both a prespecified primary analysis and subgroup or post hoc analyses, the primary analysis was extracted as the principal result and the remainder recorded separately. Discrepancies were resolved by referring back to the source publication.

Risk-of-Bias Assessment

Risk of bias was assessed independently by two reviewers. Individually randomised trials were appraised with the revised Cochrane risk-of-bias tool for randomised trials (RoB 2) across its five domains: the randomisation process, deviations from intended interventions, missing outcome data, measurement of the outcome, and selection of the reported result [10]. The two cluster-randomised trials were appraised with the cluster-randomised variant of the tool (RoB 2 CRT), which adds a sixth domain covering bias arising from the timing of identification and recruitment of individual participants in relation to the timing of cluster allocation [11]. One further trial randomised physicians rather than patients; because the units of analysis were standardised diagnostic cases specified before allocation and assigned independently of it, recruitment could not have been influenced by knowledge of the allocated arm, and the individually randomised tool was therefore applied. Non-randomised studies of interventions were appraised with the ROBINS-I tool across its seven domains: confounding, selection of participants, classification of interventions, deviations from intended interventions, missing data, measurement of outcomes, and selection of the reported result [12]. Under ROBINS-I, a judgement of low risk asserts comparability to a well-conducted randomised trial, and this threshold was applied strictly to the confounding domain. Domain-level and overall judgements were reached by consensus, with a third reviewer adjudicating where required.

Data Synthesis

The included studies differed substantially in clinical target condition, model architecture, alert recipient, comparator and outcome definition, and the point estimates reported were expressed on incompatible scales, including adjusted relative risks, hazard ratios, odds ratios, absolute risk differences and differences in medians. Statistical pooling would therefore have produced an estimate without a coherent clinical interpretation, and meta-analysis was not performed. Instead, a structured narrative synthesis was undertaken. Studies were first grouped by clinical evaluation domain and then examined within each domain for the direction and magnitude of effect on process outcomes and on patient-important outcomes separately, with the certainty of each observation interpreted in the light of the corresponding risk-of-bias judgement. Effect estimates are reported as published, with 95% confidence intervals where these were provided by the source study. Throughout, estimates derived from non-randomised designs are identified explicitly as adjusted associations rather than randomised effect estimates, so that the two are not conflated. We additionally considered a restricted synthesis of the two sepsis mortality estimates and decided against it, because one is a within-cohort comparison defined by a post-alert clinician behaviour and the other a before-and-after modelled counterfactual; pooling them would combine two different estimands rather than two estimates of a single quantity. Meta-analysis would become appropriate once at least three randomised trials of comparable systems report a common outcome on a common scale. Although formal certainty rating was not undertaken, a brief narrative certainty statement is offered for each outcome domain, informed by the GRADE domains of risk of bias, inconsistency, indirectness and imprecision [13].

Results

Study Selection

The conduct of the search, the duplicate screening process and the reasons for exclusion at each stage are reported in the Methods and are summarised quantitatively in the following text and in Figure 1; the complete database-specific search strings appear in the Appendix. The database searches identified 852 records: 274 from PubMed/MEDLINE, 223 from Embase, 162 from Scopus, 151 from Web of Science and 42 from the Cochrane Library. After 414 duplicates were removed, 438 records were screened by title and abstract, of which 328 were excluded as irrelevant. All 110 reports sought for retrieval were obtained and assessed for eligibility in full text. Of these, 101 were excluded: 41 were editorials, reviews or case reports; 32 evaluated systems that were not based on AI; and 28 were not relevant to internal medicine. Nine studies therefore satisfied all eligibility criteria and were included in the qualitative synthesis [14-22]. The study selection process is summarised in Figure 1.

**Figure 1:**
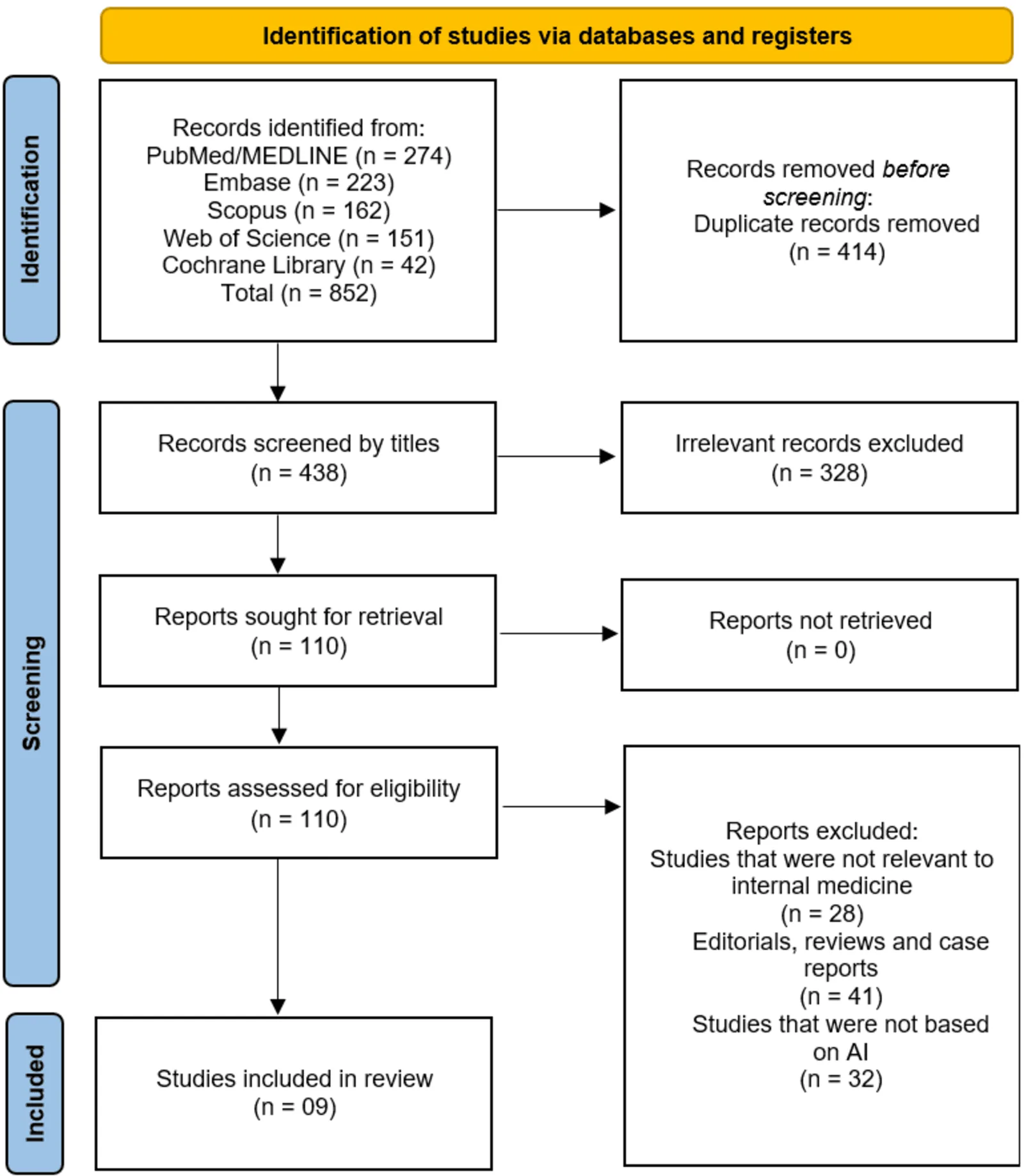
PRISMA 2020 flow diagram of study identification, screening and selection PRISMA: Preferred Reporting Items for Systematic Reviews and Meta-Analyses; AI: artificial intelligence.

Characteristics of the Included Studies

The characteristics of the nine included studies are summarised in Table 2. All were published between 2020 and 2025, reflecting the recency of prospective evaluation in this field. Six were randomised trials [15,16,19,20-22] and three were non-randomised implementation studies [14,17,18]. Among the randomised studies, two used cluster randomisation at the level of the care team or clinical shift [15,19], three randomised individual patients or patient encounters [16,20,22], and one randomised physicians [21]. The three non-randomised studies comprised a staggered multi-hospital implementation with a contemporaneous non-implemented comparison [14], a prospective multi-site cohort analysis of provider interaction with a deployed alert [17], and a before-and-after quasi-experimental study analysed with a Bayesian counterfactual model [18].

**Table 2:** Characteristics of the included studies AI: artificial intelligence; CI: confidence interval; ECG: electrocardiogram; EF: ejection fraction; EHR: electronic health record; HR: hazard ratio; ICU: intensive care unit; ML: machine learning; OR: odds ratio; PPV: positive predictive value; RR: relative risk; SOFA: Sequential Organ Failure Assessment; TREWS: Targeted Real-time Early Warning System.

Study, reference	Country	Design	Population and setting	Sample size	AI intervention	Comparator	Key findings
Escobar et al., 2020 [14]	USA	Staggered multi-hospital implementation (non-randomised)	Adults on non-ICU medical and step-down wards, 19 hospitals	43,949 alert-triggering hospitalisations	Advance Alert Monitor EHR risk model; hourly scores reviewed by remote nurses	Hospitals not yet deployed (usual care)	30-day mortality after alert lower (adjusted RR 0.84, 95% CI 0.78-0.90); 3.0 deaths avoided per 1,000 eligible patients
Yao et al., 2021 [15]	USA	Pragmatic cluster-randomised trial	Adults without prior heart failure undergoing ECG, 45 primary care sites	22,641 patients	AI-ECG flagging likely low ejection fraction, returned via the EHR	Usual care	New low EF diagnoses 2.1% vs 1.6% (OR 1.32, 95% CI 1.01-1.61); AI-positive patients 19.5% vs 14.5% (OR 1.43, 95% CI 1.08-1.91)
Tarabichi et al., 2022 [16]	USA	Randomised quality improvement initiative (patient-level)	Emergency department adults with sepsis, single centre	598 patients	ML sepsis early warning flag plus automated pharmacist notification	Standard care	Time to antibiotics 2.3 vs 3.0 h; days alive and out of hospital at 28 days 24.1 vs 22.5
Adams et al., 2022 [17]	USA	Prospective multi-site cohort study	Adults with sepsis identified by the alert, 5 hospitals	6,877 analysed (590,736 monitored)	TREWS machine learning sepsis alert requiring provider confirmation	Alert not confirmed within 3 h	Confirmation within 3 h: mortality reduced 3.3% absolute (95% CI 1.7-5.1) and 18.7% relative (95% CI 9.4-27.0); less organ failure and shorter stay
Boussina et al., 2024 [18]	USA	Before-and-after quasi-experimental study	Adults meeting Sepsis-3 criteria in 2 emergency departments	6,217 patients	COMPOSER deep learning model triggering a nurse-facing best practice advisory	Pre-intervention period (modelled counterfactual)	Mortality reduced 1.9% absolute/17% relative (95% CI 0.3-3.5); bundle compliance +5.0% (95% CI 2.4-8.0); 72-h SOFA change -4%
Kijpaisalratana et al., 2024 [19]	Thailand	Cluster-randomised trial	Emergency department adults with sepsis, tertiary hospital	574 patients	Real-time ML sepsis alert on a shared staff display	Conventional rule-based alert	Antibiotics within 1 h 68.4% vs 60.1% (P = 0.04); within 3 h 94.5% vs 89.0% (P = 0.02); median time 46 vs 50 min
Lin et al., 2024 [20]	Taiwan	Multi-site pragmatic randomised trial	Adults admitted via emergency or internal medicine departments	15,965 patients	AI-ECG mortality risk report and warning message to the responsible physician	Usual care	90-day all-cause mortality 3.6% vs 4.3%; primary endpoint met; high-risk ECG subgroup HR 0.69 (95% CI 0.53-0.90)
Goh et al., 2024 [21]	USA	Randomised trial (clinician-level)	Physicians in internal, family or emergency medicine assessing standardised cases	50 physicians; 244 cases	GPT-4 large language model in addition to conventional resources	Conventional resources alone	Diagnostic reasoning score 76% vs 74%; adjusted difference 2 percentage points (95% CI -4 to 8; P = 0.60)
Tsai et al., 2025 [22]	Taiwan	Pragmatic randomised trial	Adult inpatients under non-cardiologist care, academic centre	13,631 patients	AI-ECG real-time alert for probable ejection fraction ≤50%	Standard care	New low EF diagnoses 1.5% vs 1.1% (HR 1.50, 95% CI 1.11-2.03); PPV of echocardiography 34.2% vs 20.2%; 30-day mortality unchanged

Six studies were conducted in the United States [14-18,21], two in Taiwan [20,22] and one in Thailand [19]. Across the eight patient-level studies, 110,452 patients contributed to the primary analyses; these were drawn from far larger monitored populations, including 590,736 patients screened by the sepsis alert in one multi-site cohort [17] and 548,838 non-intensive care hospitalisations screened in another [14]. The remaining study randomised 50 attending and resident physicians trained in a general medical specialty, who completed 244 evaluable clinical case assessments [21]. Sample sizes at the level of primary analysis ranged from 574 patients [19] to 43,949 hospitalisations [14].

The included studies clustered into four evaluation domains. Four addressed sepsis recognition and treatment [16-19]; one addressed general inpatient clinical deterioration [14]; three evaluated AI-enabled electrocardiography (AI-ECG) for cardiac phenotyping or mortality risk stratification [15,20,22]; and one evaluated an LLM as an aid to physician diagnostic reasoning [21]. Model characteristics, alert delivery mechanisms, reported discrimination and effect estimates for the primary outcome of each study are presented in Table 3. Alert recipients differed materially across studies and included remote monitoring nurses [14], primary care and non-cardiology attending physicians [15,20,22], bedside and emergency department nurses [18], emergency physicians and pharmacists working in parallel [16], and emergency department clinical staff via a shared display [19].

**Table 3:** Model characteristics, alert delivery, reported performance and effect on the primary outcome AUROC: area under the receiver operating characteristic curve; CI: confidence interval; ECG: electrocardiogram; EHR: electronic health record; HR: hazard ratio; MEWS: Modified Early Warning Score; OR: odds ratio; PPV: positive predictive value; qSOFA: quick Sequential Organ Failure Assessment; RR: relative risk; SIRS: systemic inflammatory response syndrome; TREWS: Targeted Real-time Early Warning System.

Study, reference	Model class and inputs	Alert recipient and delivery	Reported model performance	Primary outcome	Effect estimate
Escobar et al., 2020 [14]	EHR-based statistical learning risk model; vital signs, laboratory values, neurologic status, severity and comorbidity indices	Hourly score to remote monitoring nurses; escalation to rapid response team	Not reported in the implementation report	30-day mortality after alert	Adjusted RR 0.84 (95% CI 0.78-0.90); favours intervention
Yao et al., 2021 [15]	Convolutional neural network applied to the 12-lead ECG waveform	Result surfaced in the EHR to the primary care team	Positive result in 6.0% of patients; discrimination not reported in the trial	New diagnosis of low ejection fraction	OR 1.32 (95% CI 1.01-1.61); favours intervention
Tarabichi et al., 2022 [16]	Proprietary EHR machine learning sepsis model; demographics, vital signs, laboratory values, orders, comorbidities	Non-interruptive provider flag plus automated pharmacist message	Alert threshold 5% predicted probability; discrimination not reported	Time to antibiotics; days alive and out of hospital	2.3 vs 3.0 h; 24.1 vs 22.5 days; favours intervention
Adams et al., 2022 [17]	TREWS machine learning early warning system trained on longitudinal EHR data	In-EHR alert requiring explicit provider confirmation	Not reported in this implementation report	In-hospital mortality	Absolute reduction 3.3% (95% CI 1.7-5.1); relative reduction 18.7% (95% CI 9.4-27.0)
Boussina et al., 2024 [18]	COMPOSER feed-forward deep neural network with conformal prediction to reject out-of-distribution inputs	Nurse-facing best practice advisory with structured acknowledgement and physician escalation	AUROC 0.938-0.945; 80% sensitivity; PPV 20.1%; 1.65 alerts per nurse per month	In-hospital mortality	Absolute reduction 1.9% (17% relative); 95% CI 0.3-3.5
Kijpaisalratana et al., 2024 [19]	Machine learning sepsis classification model using routine emergency department data	Alert on a dedicated display visible to emergency department staff	AUROC 0.93, superior to qSOFA, SIRS and MEWS	Triage-to-antibiotic time and proportion treated within 1 and 3 h	68.4% vs 60.1% within 1 h (p = 0.04); 94.5% vs 89.0% within 3 h (p = 0.02)
Lin et al., 2024 [20]	Deep learning model applied to the 12-lead ECG to predict short-term mortality risk	AI report and warning message to the responsible physician	Not reported in the trial report	All-cause mortality within 90 days	3.6% vs 4.3%; high-risk subgroup HR 0.69 (95% CI 0.53-0.90)
Goh et al., 2024 [21]	General-purpose large language model (GPT-4) with free-text clinical vignette input	Direct clinician use; no automated alerting	Not applicable	Physician diagnostic reasoning score	Difference 2 percentage points (95% CI -4 to 8); p = 0.60; no significant difference
Tsai et al., 2025 [22]	Deep learning model estimating the probability that ejection fraction is ≤50% from the 12-lead ECG	Automated on-screen alert to the attending non-cardiologist	Sensitivity 72.4%, specificity 89.1%; 9.2% flagged high risk	New low ejection fraction within 30 days	HR 1.50 (95% CI 1.11-2.03); favours intervention

Artificial Intelligence-Based Decision Support for Sepsis

In the sepsis domain, all four studies demonstrated improvements in the timeliness or completeness of sepsis care. In a single-centre randomised quality improvement initiative involving 598 emergency department patients with sepsis (285 intervention, 313 standard care), the combination of an EHR-embedded sepsis early warning flag with an automated pharmacist notification reduced the median time from arrival to antibiotic administration from 3.0 h to 2.3 h, and increased the median number of days alive and out of hospital at 28 days from 22.5 to 24.1 days [16]. A cluster-randomised trial in a Thai tertiary hospital analysing 256 intervention and 318 control patients with sepsis found that a real-time ML-assisted alert increased the proportion receiving antibiotics within 1 h from 60.1% to 68.4% (P = 0.04) and within 3 h from 89.0% to 94.5% (P = 0.02), with a modest reduction in median triage-to-antibiotic time from 50 to 46 min; the ML model achieved an AUROC of 0.93, significantly exceeding that of the quick Sequential Organ Failure Assessment, systemic inflammatory response syndrome criteria and the Modified Early Warning Score [19].

The two non-randomised sepsis studies reported effects extending to mortality. In a prospective multi-site cohort in which an ML-based targeted real-time early warning system monitored 590,736 patients across five hospitals, analysis of the 6,877 patients with sepsis identified by the alert before antibiotic initiation showed that confirmation of the alert by a provider within 3 h was associated with an adjusted absolute reduction in in-hospital mortality of 3.3% (95% CI 1.7-5.1) and an adjusted relative reduction of 18.7% (95% CI 9.4-27.0), together with reductions in organ failure and length of stay [17]. This estimate is an adjusted association conditional on provider confirmation of the alert, an exposure defined after the alert had fired and not randomly allocated, and it is not a randomised effect estimate. In a before-and-after quasi-experimental study of 6,217 septic patients across two emergency departments, deployment of a nurse-facing best practice advisory driven by a deep learning model was associated, in Bayesian causal impact analysis, with a 1.9% absolute (17% relative) reduction in in-hospital sepsis mortality (95% CI 0.3-3.5), together with improvements in bundle compliance and in the 72-hour change in Sequential Organ Failure Assessment score that are reported in full in Table 2 [18]. This too is an adjusted association derived from a modelled counterfactual rather than a randomised effect estimate. Notably, the reduction in time to antibiotics in that study was confined to alerts that nurses acknowledged with an intention to notify the physician immediately.

Artificial Intelligence-Based Decision Support for General Clinical Deterioration

The single study addressing general clinical deterioration deployed a validated automated risk model across 19 hospitals of an integrated health system in a staggered fashion, generating hourly risk scores that were reviewed remotely by trained nurses who escalated concerns to on-site rapid response teams [14]. Among 548,838 non-intensive care hospitalisations involving 326,816 patients, 43,949 hospitalisations reached the alert threshold; 15,487 occurred at sites where the programme was operational and 28,462 at sites where it had not yet been deployed. The prespecified primary outcome of this study was mortality, and it was significantly lower in the intervention cohort: mortality within 30 days of the alert fell with an adjusted relative risk of 0.84 (95% CI 0.78-0.90; P < 0.001), corresponding to 3.0 deaths avoided per 1,000 eligible patients (95% CI 1.2-4.8). This is therefore one of the four included studies reporting a mortality benefit, and, like the two non-randomised sepsis studies, it yields an adjusted association rather than a randomised effect estimate.

Artificial Intelligence-Enabled Electrocardiography

The three AI-ECG studies consistently increased the detection of clinically actionable cardiac disease, but achieved this through different mechanisms according to the care setting. In a pragmatic cluster-randomised trial across 45 primary care sites, 120 care teams comprising 358 clinicians were randomised, and 22,641 adults without prior heart failure underwent electrocardiography [15]. A positive AI-ECG result was returned in 6.0% of patients in both arms. New diagnoses of low ejection fraction increased from 1.6% to 2.1% overall (odds ratio 1.32, 95% CI 1.01-1.61; P = 0.007), with the corresponding subgroup and utilisation estimates given in Table 2. Echocardiography was obtained more often for AI-positive patients in the intervention arm, while overall echocardiogram utilisation was unchanged.

A subsequent pragmatic randomised trial applied the same concept to 13,631 hospitalised patients under the care of non-cardiologists at a Taiwanese academic centre, with 6,840 allocated to receive AI-generated ECG results and 6,791 to standard care [22]. New diagnoses of an ejection fraction of 50% or less within 30 days increased from 1.1% to 1.5% (hazard ratio 1.50, 95% CI 1.11-2.03; P = 0.023), with the high-risk subgroup estimate given in Table 2. In contrast to the primary care trial, overall echocardiography utilisation was unchanged, yet the positive predictive value of echocardiography for low ejection fraction rose from 20.2% to 34.2% (P < 0.001). Post hoc analysis indicated that the benefit was mediated by increased cardiology consultation among high-risk patients (29.3% versus 23.5%; P = 0.027) rather than by increased testing. Thirty-day all-cause mortality did not differ between groups (4.3% versus 4.5%; hazard ratio 1.00, 95% CI 0.84-1.17).

The third AI-ECG study was the only randomised trial in this review to report a mortality benefit as its primary outcome [20]. In a multi-site pragmatic randomised trial involving 15,965 patients under the care of 39 physicians, all admitted through emergency or internal medicine departments and randomised at the level of the individual patient, an AI-ECG report and warning message flagging patients predicted to be at high risk of death were delivered to the responsible physician. All-cause mortality within 90 days was 3.6% in the intervention group compared with 4.3% in the control group, and the trial met its primary endpoint. In prespecified analysis, the reduction was observed principally among patients whose electrocardiograms had been classified as high risk (hazard ratio 0.69, 95% CI 0.53-0.90).

Large Language Model-Assisted Diagnostic Reasoning

The single study evaluating an LLM as a diagnostic aid randomised 50 attending and resident physicians trained in internal medicine, family medicine or emergency medicine to use an LLM in addition to conventional resources or to use conventional resources alone, across a set of standardised diagnostic cases [21]. A total of 244 cases were completed (125 in the LLM group and 119 in the control group), with a median of five cases per participant. The median diagnostic reasoning score was 76% (interquartile range 66-87) in the LLM group and 74% (interquartile range 63-84) in the control group. In a mixed-effects model, the difference was 2 percentage points (95% CI -4 to 8; P = 0.60), and a sensitivity analysis including incomplete cases produced an equivalent result. All physicians randomised to the LLM whose transcripts were available did in fact use the tool, indicating that the null result reflected the effect of the intervention rather than non-adherence.

Risk of Bias Within Studies

Risk-of-bias judgements for the six randomised trials are presented in Table 4. Three trials were judged at low risk of bias across all applicable domains [16,20,21], two raised some concerns overall [15,19] and one was judged at high risk of bias [22]. Both trials raising some concerns were cluster-randomised and were appraised with the cluster variant of the tool. In the primary care trial, the additional domain covering the timing of participant identification relative to cluster allocation was rated as raising some concerns, because individual participants were identified only after the care teams had been allocated and identification depended on an unblinded clinician deciding to obtain an electrocardiogram, so differential identification across arms cannot be excluded [15]. In the emergency department trial, the same domain raised concerns because patients were identified during shifts that had already been allocated to the alert, and concerns were also recorded for the randomisation process and for deviations from intended interventions, reflecting allocation by clinical shift with materially unequal resulting group sizes and the unavoidably open-label nature of a shared-display alert [19].

**Table 4:** Risk-of-bias assessment of the six included randomised trials (Cochrane RoB 2 and RoB 2 CRT tools) Cluster-randomised trials are labelled as such and were appraised with the cluster variant of the tool (RoB 2 CRT); the individually randomised trials were appraised with the standard five-domain tool, for which the additional cluster domain is not applicable (NA). D1: bias arising from the randomisation process; D1b: bias arising from the timing of identification and recruitment of individual participants in relation to the timing of cluster allocation; D2: bias due to deviations from intended interventions; D3: bias due to missing outcome data; D4: bias in measurement of the outcome; D5: bias in selection of the reported result. An overall judgement of low risk requires a low-risk judgement in every applicable domain, and a high-risk judgement in any domain results in an overall high-risk judgement.

Study, reference	D1	D1b	D2	D3	D4	D5	Overall
Yao et al., 2021 [15] (cluster)	Low	Some concerns	Low	Low	Low	Low	Some concerns
Tarabichi et al., 2022 [16]	Low	Low	Low	Low	Low	Low	Low
Kijpaisalratana et al., 2024 [19] (cluster)	Some concerns	Some concerns	Some concerns	Low	Low	Low	Some concerns
Lin et al., 2024 [20]	Low	NA	Low	Low	Low	Low	Low
Goh et al., 2024 [21]	Low	NA	Low	Low	Low	Low	Low
Tsai et al., 2025 [22]	Some concerns	NA	Some concerns	Low	Low	High	High

The single high-risk judgement arose in the domain covering selection of the reported result. A documented discrepancy existed between the registered protocol and the reported analysis, including modification of the primary outcome definition and of the follow-up window, with the trial registry updated after data collection had concluded. That trial also raised some concerns regarding the randomisation process, because allocation was determined by an administrative medical record identifier rather than a concealed random sequence, and regarding deviations from intended interventions [22].

Risk-of-bias judgements for the three non-randomised studies are presented in Table 5. Two were judged at moderate risk of bias [14,18] and one at serious risk of bias [17], and in every case the determining domain was confounding. The three studies can be ranked by the design-level protection each affords against confounding. The prospective cohort study offers the least: its analysis compares patients whose sepsis alert was confirmed promptly by a provider with those whose alert was not, and prompt confirmation is itself a clinical behaviour plausibly associated with illness trajectory, diagnostic clarity and staffing levels, so residual confounding cannot be excluded and the domain was rated serious, with moderate concerns additionally recorded for selection of participants and for deviations from intended interventions [17].

**Table 5:** Risk-of-bias assessment of the three included non-randomised studies (ROBINS-I tool) The overall ROBINS-I judgement corresponds to the least favourable domain rating; a judgement of low risk asserts comparability to a well-conducted randomised trial. D1: bias due to confounding; D2: bias due to selection of participants; D3: bias in classification of interventions; D4: bias due to deviations from intended interventions; D5: bias due to missing data; D6: bias in measurement of outcomes; D7: bias in selection of the reported result.

Study, reference	D1	D2	D3	D4	D5	D6	D7	Overall
Escobar et al., 2020 [14]	Moderate	Low	Low	Low	Low	Low	Low	Moderate
Adams et al., 2022 [17]	Serious	Moderate	Low	Moderate	Low	Low	Low	Serious
Boussina et al., 2024 [18]	Moderate	Low	Low	Low	Low	Low	Low	Moderate

The remaining two studies each incorporate a structural safeguard, but neither achieves the comparability to a well-conducted randomised trial that a judgement of low risk would assert. The staggered implementation study benefited from a contemporaneous comparison group drawn from hospitals within the same integrated system that had not yet activated the programme, together with multivariable adjustment for demographic characteristics, severity of illness and comorbidity burden; however, the sequence in which hospitals activated the programme may itself have been associated with unmeasured institutional characteristics such as informatics maturity and rapid-response staffing, and the domain was therefore rated moderate [14]. The before-and-after study incorporated a Bayesian structural time-series counterfactual adjusted for emergency department volume, comorbidity burden, baseline organ dysfunction, seasonality and secular trend, but it had no concurrent control group, so unmeasured co-interventions occurring during the implementation window remain a plausible alternative explanation, and this domain was likewise rated moderate [18]. Outcome measurement was judged at low risk of bias throughout, since mortality, antibiotic timing and diagnostic coding were extracted from electronic records independently of the intervention, and missing outcome data were minimal in all nine studies.

Taken together, and interpreted informally against the GRADE domains, the certainty of the evidence differs sharply by outcome type. For process-of-care outcomes, the evidence is of moderate certainty: the direction of effect is consistent across all nine studies and derives substantially from randomised designs at low risk of bias. For patient-important outcomes, the certainty is low: three of the four mortality signals come from non-randomised designs at moderate or serious risk of bias, the single randomised mortality benefit was concentrated in a subgroup, and the evidence is confined to two clinical domains in three countries.

Discussion

This systematic review identified nine original studies in which an AI-based CDSS was prospectively deployed within adult internal medicine or acute general medical care and evaluated against a defined comparator. Three principal findings emerge. First, the evidence base is small, recent and geographically concentrated: nine studies published across six years, all from three countries, with more than half from a single health system context. Second, there is a consistent and reasonably robust signal that these systems improve the processes of care they are designed to influence, including the timeliness of antibiotic administration, adherence to sepsis bundles, the detection of previously undiagnosed left ventricular systolic dysfunction and the diagnostic yield of confirmatory testing. Third, and considerably more tentatively, the evidence that this process improvement translates into improved patient-important outcomes rests on four studies, of which three were non-randomised [14,17,18] and only one was randomised [20]. The three non-randomised studies yield adjusted associations rather than randomised effect estimates, and in the single randomised trial in which mortality was the primary endpoint, the benefit was concentrated in an algorithm-defined high-risk subgroup [20]. This gradient, from process evidence resting largely on randomised designs to mortality evidence resting largely on non-randomised ones, is the central finding of this review and should temper the enthusiasm with which these technologies are currently being adopted. It is also worth stating plainly how these systems are thought to act. They do not improve outcomes by predicting more accurately in the abstract; they improve outcomes only insofar as the prediction changes a specific downstream clinician behaviour, and the operative behaviour differs by system: earlier antibiotic administration in the sepsis studies, escalation to a rapid response team in the deterioration study, and cardiology consultation or confirmatory echocardiography in the electrocardiography studies.

The concentration of evidence in sepsis is readily explained. Sepsis has a well-defined time-critical treatment window, a plausible causal chain linking earlier recognition to earlier antibiotics and thence to survival, and an existing quality infrastructure that already measures bundle compliance and time to antibiotics. It is therefore the condition in which an early warning algorithm is most likely both to change behaviour and to have that change captured. Even here, however, the mortality evidence rests on non-randomised designs, and the two studies differ importantly in their vulnerability to bias. The prospective cohort analysis of a targeted real-time early warning system reported an adjusted 18.7% relative reduction in in-hospital mortality among patients whose alerts were confirmed within 3 h [17], but its comparison is between patients whose care teams responded promptly and those whose teams did not. Clinician responsiveness is not randomly distributed. It is plausibly greater for patients who are more obviously unwell, more clearly infected, or cared for on better-staffed shifts. Each of these is independently associated with outcome, which is why this study was rated at serious risk of bias for confounding. The before-and-after evaluation of a deep learning sepsis model reported a comparable 17% relative mortality reduction using an explicit counterfactual model adjusted for case-mix, volume, seasonality and secular trend [18]. That modelling is a genuine methodological strength. It cannot, however, substitute for a concurrent control group, and unmeasured co-interventions occurring during the implementation window remain a plausible alternative explanation, which is why this study was rated at moderate rather than low risk of bias for confounding. The same reasoning applies to the deterioration study, whose staggered activation sequence across 19 hospitals may itself have tracked unmeasured institutional characteristics [14]. The randomised evidence within this domain is more modest in its claims: the two randomised sepsis trials demonstrated faster antibiotics and, in one case, more days alive and out of hospital, but neither was powered for mortality [16,19].

Placing these findings in the context of the earlier literature clarifies how much has and has not changed. An early randomised trial of an ML-based severe sepsis prediction algorithm in a small intensive care population reported reductions in in-hospital mortality and length of stay, but enrolled only 142 patients and was conducted in a single centre, and its estimates were correspondingly imprecise [23]. In the intervening years, the field has largely occupied itself with model derivation rather than deployment. The landmark demonstration that a convolutional neural network applied to a standard 12-lead electrocardiogram could identify asymptomatic left ventricular systolic dysfunction with an area under the curve of 0.93 [24] was preceded by two years by the first pragmatic randomised trial testing whether returning that output to a physician changed anything [15]. The interval between a well-validated model and its first prospective clinical evaluation is therefore measured in years, and for the majority of published algorithms it has not yet elapsed at all. The present review, in identifying only nine such evaluations in the whole of internal medicine, quantifies the scale of that deficit.

A recurring theme across the included studies is that model discrimination is a poor predictor of clinical impact. The most instructive counterexample lies just outside this review: an external validation of a widely implemented proprietary sepsis prediction model, deployed at hundreds of hospitals, found poor discrimination and calibration, generating alerts on a large proportion of hospitalised patients while missing most sepsis cases [25]. Subsequent work demonstrated that the same proprietary model's performance varied markedly across nine networked hospitals within a single health system, indicating that discrimination is not a transportable property of a model but a property of the model in a particular data environment [26]. Two of the studies in the present review illustrate the corollary. The deep learning sepsis model evaluated in the before-and-after study was explicitly engineered to suppress false alarms by rejecting out-of-distribution inputs, and generated only 1.65 alerts per nurse per month; fewer than 6% of alerts were dismissed without acknowledgement [18]. The ML alert evaluated in the Thai cluster-randomised trial outperformed all three conventional screening tools it was compared against [19]. In both cases, the design choices that shaped the alert burden and the credibility of the alert appear at least as consequential as the model architecture.

The AI-ECG studies provide the clearest illustration that identical algorithmic outputs produce different clinical effects in different care contexts. In primary care, returning a positive AI-ECG result increased echocardiography ordering for flagged patients by more than 11 percentage points and increased new diagnoses of low ejection fraction, with no change in overall imaging utilisation [15]. In hospitalised patients under non-cardiologist care, the same conceptual intervention increased new diagnoses by a comparable relative margin, but did so without any increase in echocardiography. Instead, the positive predictive value of echocardiography rose from 20.2% to 34.2%. The mechanism appeared to be a shift towards cardiology consultation for flagged patients rather than an increase in testing volume [22]. In other words, in a resource-rich inpatient environment where diagnostic vigilance is already high, the algorithm functioned less as a prompt to test more and more as a prompt to test the right patients. This has direct implications for implementation planning: the outcome that a health system should expect from an AI-ECG deployment, and therefore the metric by which it should evaluate it, depends on the baseline diagnostic behaviour of the setting into which it is introduced. A deployment evaluated solely on test ordering volume would have been judged a failure in the inpatient setting despite achieving its diagnostic objective.

The null result of the LLM trial deserves particular attention, given the speed with which such tools are entering clinical workflows. Physicians randomised to use an LLM alongside conventional resources achieved diagnostic reasoning scores no better than those using conventional resources alone, with a point estimate of 2 percentage points and a confidence interval excluding any clinically meaningful benefit [21]. Adherence was complete, so the finding cannot be attributed to non-use, and the trial was judged at low risk of bias across all domains, so the null result is unlikely to be an artefact of methodological weakness. The most parsimonious interpretation is that access to a capable model is not the same as effective human-model collaboration, and that physicians in this study did not extract the model's full capability, whether because of the way queries were framed, because of appropriate scepticism towards outputs that conflicted with their own reasoning, or because the interface did not support integration of the model's output into a structured differential. This finding argues strongly that the human-computer interaction layer, rather than raw model capability, is now the rate-limiting step for this class of decision support, and that trials of LLMs in clinical settings must evaluate the whole sociotechnical system rather than the model in isolation.

Several important knowledge gaps follow from these observations. The most conspicuous is the near-total absence of evidence in chronic disease management, which constitutes the majority of internal medicine practice. No eligible study addressed the ambulatory management of diabetes, chronic kidney disease, chronic obstructive pulmonary disease, chronic liver disease, inflammatory rheumatological conditions or the longitudinal management of heart failure, despite a large volume of retrospective modelling in each of these areas. The evidence is also geographically narrow. All nine studies originated in the United States, Taiwan or Thailand, with none from Europe, South Asia, the Middle East, Latin America or Africa. This matters because model performance is data-environment dependent, and because algorithms trained on one population may systematically underperform in another. The demonstration that a widely deployed population health algorithm substantially underestimated the illness burden of Black patients, because it used healthcare expenditure as a proxy for need, remains the definitive illustration that an algorithm can be technically well-calibrated to its training label while being profoundly miscalibrated to the clinical question it is used to answer [27]. Only one of the nine included studies reported effects stratified by race or ethnicity in any detail, and none prespecified an equity analysis.

Methodological gaps are equally pressing. None of the included studies reported a formal economic evaluation, despite the fact that decisions about deploying these systems are made by health systems weighing licensing, integration and maintenance costs against uncertain benefit. Harms were reported inconsistently and usually informally: alert fatigue, antibiotic overuse and downstream investigation cascades were addressed narratively in some studies and not at all in others, and no study prespecified adverse consequences as an outcome. Reporting against AI-specific standards was variable. The CONSORT-AI extension specifies items including algorithm version, input data handling, human-AI interaction and the management of poor-quality inputs [7], and the DECIDE-AI guideline provides a corresponding framework for the early-stage clinical evaluation of AI-driven decision support, covering the implementation context, user training, safety monitoring and human factors [28]. Only two of the six randomised trials explicitly reported adherence to CONSORT-AI, and none reported against DECIDE-AI. Finally, no included study reported a prespecified plan for post-deployment model monitoring in its primary publication, although one described a data quality dashboard, biweekly performance review and a predetermined change control plan for retraining [18]. Given that calibration drift is an expected rather than exceptional behaviour of deployed models, the absence of monitoring protocols represents a material gap between how these systems are evaluated and how they must be governed once adopted.

The research agenda that follows is reasonably clear. Multicentre randomised trials with patient-important primary endpoints are required, powered adequately for mortality or a composite of mortality and organ dysfunction rather than for process surrogates. Such trials should prespecify the mechanism through which benefit is expected to operate, since the AI-ECG studies demonstrate that the operative pathway differs by setting, and should measure that mechanism directly. They should report harms with the same rigour as benefits, incorporate concurrent economic evaluation, and adhere explicitly to CONSORT-AI and DECIDE-AI. Trials should extend beyond the acute time-critical conditions that currently dominate the literature into the chronic disease domains where the burden of internal medicine practice actually lies, and should be conducted in health systems with differing baseline resource levels and differing patient populations so that transportability can be assessed rather than assumed. Until such evidence accumulates, the appropriate posture for clinicians and health systems is one of cautious, monitored adoption: these systems demonstrably change what clinicians do, which is a genuine achievement, but the evidence that they change what happens to patients remains, on the present synthesis, considerably thinner than the intensity of current interest would suggest.

Limitations

This review has several limitations. The requirement for prospective deployment within a live clinical workflow excluded a large body of retrospective modelling work, so the review describes only the leading edge of a much larger literature, and the boundary of internal medicine is not sharply defined, so the decision to include emergency department studies of undifferentiated adult medical presentations and one clinician-randomised trial is defensible but not the only reasonable position. The review was not prospectively registered, searches were limited to five databases, and grey literature, trial registries and preprints were not searched, so unpublished or negative implementation evaluations may be under-represented and publication bias is a realistic concern that the small number of included studies precluded assessing statistically. Meta-analysis was not undertaken because of heterogeneity in conditions, models, comparators and outcome scales, so no pooled estimate is offered, and certainty of evidence was not formally graded using GRADE. Finally, one randomised trial was at high risk of bias, two raised some concerns, and of the non-randomised studies two were at moderate and one at serious risk of bias; because three of the four mortality signals derive from non-randomised designs and are therefore adjusted associations rather than randomised effect estimates, conclusions regarding patient-important outcomes remain provisional.

## Conclusions

Prospectively deployed AI-based CDSSs in adult internal medicine improve the processes of care they target with reasonable consistency, shortening time to antibiotics, increasing sepsis bundle compliance, raising the detection of previously undiagnosed left ventricular systolic dysfunction and improving the diagnostic yield of confirmatory testing; evidence that these gains extend to mortality is more limited, deriving from four studies of which three were non-randomised and therefore yield adjusted associations rather than randomised effect estimates, and in which the single randomised mortality benefit was concentrated in an algorithm-defined high-risk subgroup. Clinical benefit appears to be determined less by a model's discriminative performance than by how its output is routed, presented, acknowledged and acted upon within a specific clinical environment, and identical algorithms produced different effects in different care settings. Given that the evidence base is small, recent, restricted to three countries and almost entirely confined to acute time-critical conditions, adequately powered multicentre randomised trials with patient-important primary endpoints, prespecified harm and equity analyses, concurrent economic evaluation, explicit post-deployment monitoring plans and adherence to AI-specific reporting standards are required before routine adoption across internal medicine can be recommended based on demonstrated patient benefit.

## Appendices

**Table 6:** Full database-specific search strategies

Database (platform)	Date searched	Search strategy (query)	Limits/Filters	Records retrieved
MEDLINE (PubMed)	Database inception to 31 May 2026	#1 "artificial intelligence"[MeSH] OR "machine learning"[MeSH] OR "deep learning"[MeSH] OR "neural networks, computer"[MeSH] OR "artificial intelligence"[tiab] OR "machine learning"[tiab] OR "deep learning"[tiab] OR "neural network"[tiab] OR "large language model"[tiab] OR "predictive analytic"[tiab] OR "computer-aided"[tiab] OR algorithm[tiab] #2 "decision support systems, clinical"[MeSH] OR "decision support techniques"[MeSH] OR "clinical decision support"[tiab] OR "decision support system"[tiab] OR "early warning system"[tiab] OR "early warning score"[tiab] OR "best practice advisory"[tiab] OR "electronic alert"[tiab] OR "clinical alert"[tiab] OR "risk score"[tiab] OR "predictive model"[tiab] #3 "internal medicine"[MeSH] OR "hospital medicine"[MeSH] OR "hospitalists"[MeSH] OR "internal medicine"[tiab] OR "general medicine"[tiab] OR "hospital medicine"[tiab] OR inpatient[tiab] OR hospitali[tiab] OR "medical ward"[tiab] OR "general ward"[tiab] OR "emergency department"[tiab] OR sepsis[MeSH] OR sepsis[tiab] OR "septic shock"[tiab] OR "heart failure"[MeSH] OR "heart failure"[tiab] OR "clinical deterioration"[MeSH] OR "clinical deterioration"[tiab] OR "ejection fraction"[tiab] OR diagnos[tiab] #4 #1 AND #2 AND #3 #5 #4 NOT ("review"[Publication Type] OR "systematic review"[Publication Type] OR "meta-analysis"[Publication Type] OR "editorial"[Publication Type] OR "letter"[Publication Type] OR "comment"[Publication Type] OR "case reports"[Publication Type])	No language or geographic restriction. Fields: [MeSH] = Medical Subject Headings; [tiab] = title/abstract. Excluded review articles, editorials, letters, comments, and case reports.	274
Embase (Elsevier, Emtree)	Database inception to 31 May 2026	#1 'artificial intelligence'/exp OR 'machine learning'/exp OR 'deep learning'/exp OR 'artificial neural network'/exp OR ('artificial intelligence' OR 'machine learning' OR 'deep learning' OR 'neural network' OR 'large language model' OR 'predictive analytic' OR algorithm):ti,ab,kw #2 'clinical decision support system'/exp OR 'decision support system'/exp OR ('clinical decision support' OR 'decision support system' OR 'early warning system' OR 'early warning score' OR 'best practice advisory' OR 'electronic alert' OR 'risk score'):ti,ab,kw #3 'internal medicine'/exp OR 'hospital medicine'/exp OR 'sepsis'/exp OR 'heart failure'/exp OR 'clinical deterioration'/exp OR ('internal medicine' OR 'general medicine' OR inpatient OR hospitali* OR 'medical ward*' OR 'emergency department' OR sepsis OR 'septic shock' OR 'heart failure' OR 'ejection fraction' OR 'clinical deterioration'):ti,ab,kw #4 #1 AND #2 AND #3 #5 #4 NOT ('review'/it OR 'editorial'/it OR 'letter'/it OR 'note'/it OR 'conference abstract'/it OR 'conference paper'/it OR 'short survey'/it)	No language or geographic restriction. /exp = exploded Emtree term; :ti,ab,kw = title, abstract, keyword. Excluded reviews, editorials, letters, notes, conference abstracts/papers, and short surveys.	223
Scopus	Database inception to 31 May 2026	TITLE-ABS-KEY ( "artificial intelligence" OR "machine learning" OR "deep learning" OR "neural network" OR "large language model" OR "predictive analytic" OR algorithm ) AND TITLE-ABS-KEY ( "clinical decision support" OR "decision support system" OR "early warning system" OR "early warning score" OR "best practice advisory" OR "electronic alert" OR "risk score" ) AND TITLE-ABS-KEY ( "internal medicine" OR "general medicine" OR "hospital medicine" OR inpatient OR hospitali* OR "medical ward*" OR "emergency department" OR sepsis OR "septic shock" OR "heart failure" OR "clinical deterioration" OR "ejection fraction" )	No language or geographic restriction. TITLE-ABS-KEY searches title, abstract, and keywords. DOCTYPE ar = article. Limited to articles only (DOCTYPE "ar") and to subject areas MEDI (Medicine) and HEAL (Health Sciences).	162
Web of Science Core Collection	Database inception to 31 May 2026	#1 TS = ("artificial intelligence" OR "machine learning" OR "deep learning" OR "neural network" OR "large language model" OR "predictive analytic" OR algorithm) #2 TS = ("clinical decision support" OR "decision support system" OR "early warning system" OR "early warning score" OR "best practice advisory" OR "electronic alert" OR "risk score") #3 TS = ("internal medicine" OR "general medicine" OR "hospital medicine" OR inpatient OR hospitali* OR "medical ward*" OR "emergency department" OR sepsis OR "septic shock" OR "heart failure" OR "clinical deterioration" OR "ejection fraction") #4 #1 AND #2 AND #3 #5 #4, refined by Document Types: ARTICLE; Web of Science Categories: MEDICINE GENERAL INTERNAL OR HEALTH CARE SCIENCES SERVICES OR MEDICAL INFORMATICS OR CRITICAL CARE MEDICINE	No language or geographic restriction. TS = topic (title, abstract, author keywords, Keywords Plus). Limited to articles only and to specific Web of Science categories.	151
Cochrane Library (CENTRAL)	Database inception to 31 May 2026	#1 MeSH descriptor: [Artificial Intelligence] explode all trees #2 ("artificial intelligence" OR "machine learning" OR "deep learning" OR "neural network" OR "large language model" OR algorithm):ti,ab,kw #3 #1 OR #2 #4 MeSH descriptor: [Decision Support Systems, Clinical] explode all trees #5 ("clinical decision support" OR "decision support system" OR "early warning system" OR "electronic alert" OR "risk score"):ti,ab,kw #6 #4 OR #5 #7 ("internal medicine" OR "general medicine" OR inpatient OR hospitali* OR "medical ward*" OR "emergency department" OR sepsis OR "heart failure" OR "clinical deterioration"):ti,ab,kw #8 #3 AND #6 AND #7	No language or geographic restriction. MeSH descriptors exploded; ti,ab,kw = title, abstract, keyword. Limited to trials only.	42
Supplementary searching	N/A	Reference lists of all nine included articles and of all relevant reviews identified during screening were hand-searched. Forward citation tracking of the included articles was performed in Scopus and Google Scholar. No additional eligible reports were identified through supplementary searching.	Records from all sources were exported to EndNote 21 for deduplication and screened in Rayyan by two independent reviewers.	0
